# Interpretable clinical prediction via attention-based neural network

**DOI:** 10.1186/s12911-020-1110-7

**Published:** 2020-07-09

**Authors:** Peipei Chen, Wei Dong, Jinliang Wang, Xudong Lu, Uzay Kaymak, Zhengxing Huang

**Affiliations:** 1 College of Biomedical Engineering and Instrumental Science, Zhejiang University, Hangzhou, China; 2 School of Industrial Engineering, Eindhoven University of Technology, Eindhoven, The Netherlands; 3 Department of Cardiology, Chinese PLA General Hospital, Beijing, China; 4 Cardiocloud medical technology, Beijing, China

**Keywords:** Interpretability, Attention mechanism, Clinical prediction, Deep learning

## Abstract

**Background:**

The interpretability of results predicted by the machine learning models is vital, especially in the critical fields like healthcare. With the increasingly adoption of electronic healthcare records (EHR) by the medical organizations in the last decade, which accumulated abundant electronic patient data, neural networks or deep learning techniques are gradually being applied to clinical tasks by utilizing the huge potential of EHR data. However, typical deep learning models are black-boxes, which are not transparent and the prediction outcomes of which are difficult to interpret.

**Methods:**

To remedy this limitation, we propose an attention neural network model for interpretable clinical prediction. In detail, the proposed model employs an attention mechanism to capture critical/essential features with their attention signals on the prediction results, such that the predictions generated by the neural network model can be interpretable.

**Results:**

We evaluate our proposed model on a real-world clinical dataset consisting of 736 samples to predict readmissions for heart failure patients. The performance of the proposed model achieved 66.7 and 69.1% in terms of accuracy and AUC, respectively, and outperformed the baseline models. Besides, we displayed patient-specific attention weights, which can not only help clinicians understand the prediction outcomes, but also assist them to select individualized treatment strategies or intervention plans.

**Conclusions:**

The experimental results demonstrate that the proposed model can improve both the prediction performance and interpretability by equipping the model with an attention mechanism.

## Background

Recently, deep learning techniques have achieved great success in healthcare domain due to their impressive performance [1–3]. Specifically, with the increasingly adoption of electronic healthcare records (EHR) by the medical organizations in the last decade, a large volume of electronic patient data is accumulated, and thereafter neural networks or deep learning techniques are gradually being applied to clinical prediction tasks by utilizing the huge potential of EHR data, e.g. clinical risk assessment, outcome prediction, treatment effect estimation and treatment recommendations [4–7].

However, typical deep learning models are black-boxes, which are not transparent and the prediction outcomes of which are difficult to interpret [8]. Therefore, although deep learning models have shown remarkable performance on most clinical prediction tasks, the lack of interpretability makes them difficult to be practically adopted in the real clinical settings.

The interpretability is vital for the successful applications of machine learning models in the healthcare domain [9]. The reasons for the requirement of interpretable models are multiple, as indicated in literature [10, 11]. Firstly, interpretability is the prerequisite for trust [12]. Healthcare professionals tend to have more confidence in the models which are well-understood or the models which can provide explanations. Secondly, interpretable models are expected to provide useful information to healthcare professionals and assist them to make decisions [13]. For example, instance-based models can support clinicians to take actions by pointing to similar patients. Additionally, interpretable models can help healthcare professionals gain insights into new knowledge [14]. There are also other reasons for the requirement of interpretability, such as legislation (the right to explanation in EU’s GDPR (General Data Protection Regulation)) [15], reducing bias and capturing causality [16].

In this study, we present an attention based neural network model to improve the interpretability of the clinical predictions. We evaluated our model on a real-world EHR dataset to predict the readmissions of heart failure patients. The experimental results demonstrate that our proposed model can not only improve the prediction performance but also provide interpretations on the prediction results.

### Related works

In this section, we briefly review the existing interpretable models or techniques enabling interpretations for black-box deep learning models, which can be broadly classified into two categories [10].

The first category relates to transparency of the model (i.e. how does the model work?). A transparent model can be understood at the level of the model itself, i.e. mechanistic or algorithmic transparency [17]. Linear model or logistic regression, decision tree and rule based models (e.g. fuzzy inference system [18]) are commonly considered to be transparent [8]. For example, the coefficients of the linear model could be interpreted as the strengths of the relationship between each feature and the label, and the sign of each coefficient indicates the direction of the relationship. However, such models become less interpretable when the models are too complex, e.g. deep decision trees and unmanageable number of rules [10].

The second category comprises various techniques which can provide post-hoc explanations for the black-box models. In contrast to the intrinsically transparent models in the first category mentioned above, the post-hoc interpretability may not attempt to interpret the inner work of the model, but seek to explain the predictions of the opaque or black-box model without sacrificing the performance [8, 10]. The popular techniques of post-hoc interpretations contain explanations by text (natural language), explanations by visualization, explanations by a surrogate transparent model, and explanations by attention mechanism, as briefly discussed below:

Text explanations can provide qualitative understanding of the model predictions by presenting human understandable verbal words. One approach is to train two models simultaneously, one for prediction and another to generate textual explanations. For example, McAuley and Leskovec [19] presented a model to recommend products by simultaneously training a latent factor model for rating prediction and a topic model for textual product reviews. The predicted ratings can be explained by the top words in the topics.

Visualization explanations (e.g. heat maps) can provide post-hoc interpretations by visualizing what the model learned. For instance, t-SNE (t-Distributed Stochastic Neighbor Embedding) is commonly exploited to visualize the learned high-dimensional representations in 2D space [20].

Explanations by surrogate model improve the interpretability of black-box models by interpreting the source opaque model utilizing a transparent surrogate model (e.g. linear model, logistic regression, decision tree, instance-based model or rule-based model). For example, after training a deep learning model, we can identify the most similar patients to the source patient based on the learned latent representations to justify the model prediction [21, 22]. In addition, LIME (Local Interpretable Model-agnostic Explanations) [11] explain the predictions of an opaque model by approximating it locally with an interpretable model, e.g. learning an interpretable model locally around the prediction.

Attention explanations are recently advocated to open a new window for interpreting deep learning models. Originally, the attention mechanism is mainly used to model dependencies between sequences regardless of their actual distances [23, 24]. It has achieved great success in many sequence modeling tasks, e.g. neural machine translation [23] and speech recognition [25]. Recently, attention mechanisms are increasingly applied to improve not only the accuracy but also the interpretability of deep learning models [26–28]. In [26], the authors proposed the GRaph-based Attention Model (GRAM) for healthcare representation learning, which infuses information from medical ontologies into deep learning models via attention mechanism and the attention behavior during prediction could be explained intuitively by showing the attention weights of each node in the knowledge graph. Choi et.al [27] proposed a model known as RETAIN, a two-level neural attention model for sequential data, which provides detailed interpretation of the prediction results while retaining the prediction accuracy comparable to RNN. In RETAIN, when keeping the attention fixed, the model prediction can be interpreted by analyzing the changes of each label in relation to changes in the original inputs, i.e. the input variable that yields the largest change in label will be the input variable with highest contribution.

Along with this direction, this study proposes an interpretable neural model equipped with an attention mechanism to address the clinical prediction problem, which can provide patient-specific attention weights on features such that the prediction results can be explained.

## Methods

In this section, we firstly introduce the problem definition and notations used in this paper, and then present our proposed model in detail.

### Problem definition

In this paper, the dataset is extracted from a large amount of EHR. A particular patient sample contains *m* features (characteristics) and is usually represented as a feature vector *x*. The dataset consisting of *n* patient samples can be represented as a matrix:

1 $$ X=\left[{x}_1,\cdots, {x}_n\right]=\left[\underset{x_{1m}}{\overset{x_{11}}{\vdots }}\kern0.24em \underset{\cdots }{\overset{\cdots }{\ddots }}\kern0.24em \underset{x_{nm}}{\overset{x_{n1}}{\vdots }}\right] $$

Let *Y* be the labels (i.e. readmitted/non readmitted) of the *n* patient samples and can be denoted as:

2 $$ Y=\left[{y}_1,\cdots, {y}_n\right] $$

The goal of this study is to predict the labels of the patients based on the patient characteristics in an interpretable manner.

### The proposed model

**Fig. 1:**
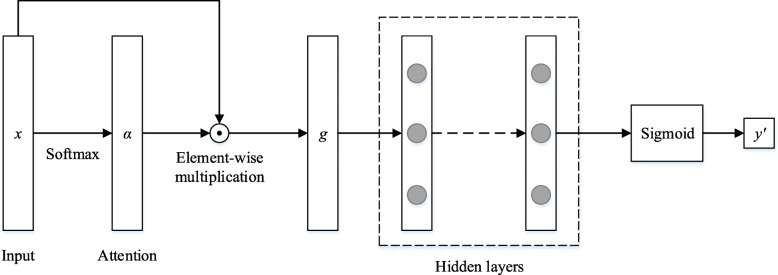
The overview of the proposed model

Figure 1 depicts the overview of the proposed attention-based model (we denote it as MLP_attention). The core idea of the proposed model is to use an attention mechanism to capture the contribution of each input patient feature to the prediction, so that the generated prediction results can be interpreted. We introduce our proposed model below in detail.

Given the inputs *x*, we firstly feed *x* into a fully connected layer (with *Softmax* function as the activation function) to generate the attention signals *α* (see Eq. (3)). The number of output nodes of the fully connected layer is the same with the length of *x*, i.e. *m*. The *Softmax* function ensures that the sum of the attention weights of all patient features equals to one.

3 $$ \alpha = Soft\max\;\left( Wx+b\right) $$

Then, we obtain the final input representation *g* by the element-wise multiplication of the inputs *x* and the generated attention signals *α*:

4 $$ g=\alpha \odot x $$

where ⨀ stands for the element-wise multiplication.

With the final input representation *g*, a multilayer perceptron (MLP) model with several hidden layers is used to predict the labels. The function of the output layer is *Sigmoid* function. We select 0.5 as the threshold to obtain the predicted labels.

We train the attention layer together with MLP. The loss function of the proposed model is defined as the follows:

5 $$ L=-\frac{1}{n}{\sum}_{i=1}^n\left({y}_i\log {y}_i^{\hbox{'}}+\left(1-{y}_i\right)\log \left(1-{y}_i^{\hbox{'}}\right)\right)+\lambda {\left|\left|\Theta \right|\right|}_2^2 $$

where ${y}_i^{\prime }$ is the predicted label for patient *i*, *λ* is the weight parameter to balance two losses.

## Experiments and results

### Dataset and experimental settings

In this study, we evaluate our proposed model on a real-world clinical dataset consisting of 736 heart failure (HF) patients collected from the Cardiology Department at the Chinese PLA General Hospital. The objective of the experiments is to predict readmissions within 1 year based on the patient characteristics. Specifically, each patient contains 105 features, including demographics (e.g. age and gender), vital signs (e.g. blood pressure and heart rate), lab tests (e.g. NT-proBNP and CTnT), echocardiography (e.g. ejection fraction and QRS interval), comorbidities (e.g. diabetes and renal insufficiency), length of stay (LOS) and medications (e.g. ACEI/ARB, beta blocker and MRA). These patients were followed up for 1 year to check the readmissions within 1 year (461 readmitted, 275 not readmitted). Patient features with more than 30% missing values were not included in this work, while the features with less than 30% missing values were imputed by the median of the features.

Note that a prior ethics approval was obtained from the data protection committee and the institutional review board of the hospital, and the patient data was anonymized in our study.

We compared the performance of the proposed model with three baseline models: logistic regression (LR), MLP (without the attention mechanism) and stacked denoising auto-encoder (SDAE). For both the proposed and the baseline models, we employed the five-fold cross-validation strategy on 80% of the data for training and tuning the model, and evaluated the performance of the trained model on the rest 20% of the data (final test set) that was not used during the training process. The experiments were repeated ten times and the final performances were averaged on the ten repetitions. Accuracy, precision, recall, F1 score and AUC (Area under ROC curve) were employed as the evaluation metrics.

In terms of the hyper-parameter settings, the learning rate is 0.001, the L2 coefficient *λ* is 0.001. The numbers of hidden layers of were tested from one to five.

### Prediction performance

**Fig. 2:**
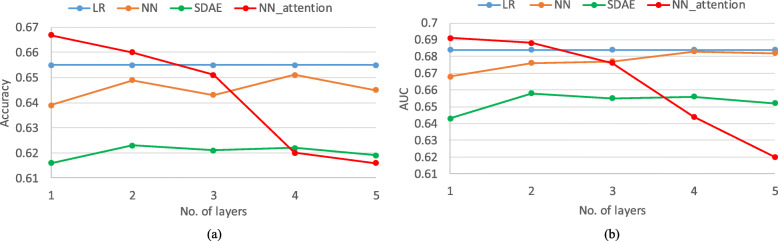
The (**a**) Accuracy and (**b**) AUC of the proposed model with different number of hidden layers in comparison with baseline models

We firstly investigated the influence of the number of hidden layers of the proposed model on the performance of readmission prediction in terms of both accuracy and AUC, as illustrated in Fig. 2. The results show that the proposed model achieved the best performance in terms of both accuracy and AUC among the four models. With the number of hidden layers increasing, the performance of the proposed model decreased. For MLP, both accuracy and AUC reached the peak when there were four hidden layers, and began to drop when the number of hidden of layers increased to five probably because of over-fitting. SADE achieved the best performance with two encoding and decoding layers.

Table 1 records the performance of the proposed model (one hidden layer) and the three baseline models LR, MLP (four hidden layers) and SDAE (two encoding and decoding layers). It indicated that although MLP performed the best in terms of recall, the proposed model outperformed MLP, LR and SDAE in terms of accuracy, precision, F1 score and AUC. Specifically, with the attention mechanism, the proposed model had 2.5, 2.6, 1.1 and 1.2% performance gain compared to MLP in terms of accuracy, precision, F1 score and AUC respectively. This demonstrates that the attention mechanism can improve the performance of MLP.

**Table 1:** The prediction performance of all the models (mean ± std. (standard deviation))

Models	Accuracy	Precision	Recall	F1	AUC
MLP_attention	**0.667 ± 0.030**	**0.710 ± 0.020**	0.795 ± 0.059	**0.749 ± 0.029**	**0.691 ± 0.047**
MLP	0.651 ± 0.028	0.692 ± 0.022	**0.799 ± 0.062**	0.741 ± 0.030	0.683 ± 0.041
LR	0.655 ± 0.027	0.700 ± 0.019	0.792 ± 0.043	0.743 ± 0.024	0.684 ± 0.039
SDAE	0.623 ± 0.025	0.670 ± 0.018	0.782 ± 0.038	0.722 ± 0.022	0.658 ± 0.033

To examine the statistical difference of the performances between the proposed model and the baseline models, we conducted the paired-samples t-test for each pair of models. The paired sample t-test is a statistical procedure used to determine whether the mean difference between two sets of observations is zero [29]. In our study, the proposed model and the baseline models predicted the labels (readmitted/non-readmitted) of all patient samples in the test sets and resulted in paired sets of observations. As can be seen in Table 2, the predicted labels of each pair of models are statistically different (*p* < 0.01).

**Table 2:** The *p*-value of paired t-test between the proposed model and baseline models

Models	MLP_attention	MLP	LR	SDAE
MLP_attention	–	0.003	0.005	0.0008
MLP		–	0.009	0.005
LR			–	0.003
SDAE				–

### Attention analysis

**Fig. 3:**
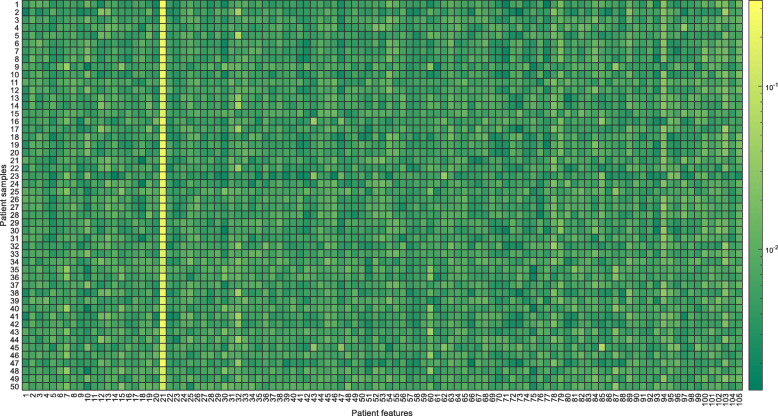
The heat map showing the contribution (attention weight) of each feature for readmission identified by the proposed model for 50 randomly selected patients

The interpretability of the generated prediction results is significantly important in healthcare. Since the proposed model is based on the attention mechanism, it is easy to obtain the contribution of each patient feature by the attention weights. Fig. 3 shows the heat map of the contribution (i.e., the attention weight) of each feature for readmission identified by the proposed model for 50 randomly selected patients. Each row is a patient sample and each column is a feature. Note that the 105 features are denoted as serial numbers in the heat map for clearness. The color in the heat map corresponds to the patient feature contribution (i.e. the log value of attention weight). From Fig. 3, we can observe that *NT-proBNP* (the 21st feature) got the most attention in almost all the patients. This observation is in accordance with the clinical practice, in which *NT-proBNP* is an essential risk factor for heart failure patients [30].

**Fig. 4:**
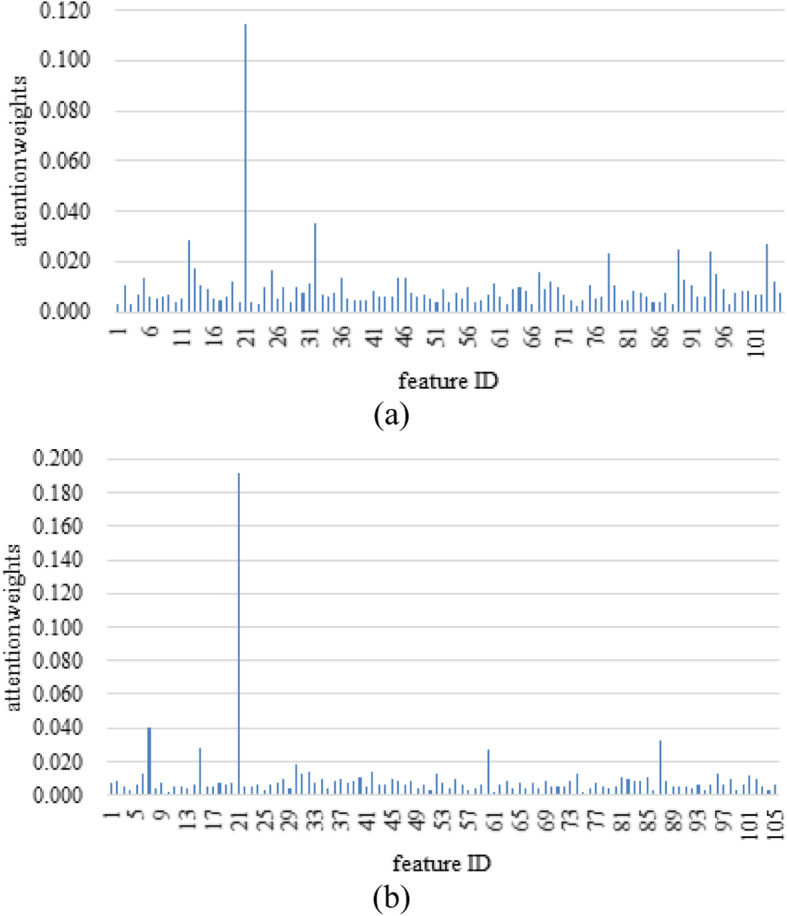
The bar plots of the attention weights of each patient feature for two randomly selected patients

Additionally, we can observe the personalized risk factors for each patient besides *NT-proBNP*. For example, we present two heart failure patients with visualizing their relating predictors in Fig. 4. As we can see the first patient in Fig. 4a, *Sodium* (the 32nd feature) and *CHD (Coronary Heart Disease)* (the 12th feature) are the next two most important predictors, whilst for patient 2 in Fig. 4b, the next two most important predictors are *SBP (Systolic blood pressure)* (the 7th feature) and *Left ventricular end-systolic volume* (the 87th feature). This shows that heart failure patient may have different subtypes, i.e. personalized profiles with individualized risk factors. In Table 3, we listed the top-ranked features (attention weights> 0.2) of these two patients respectively. As the clinical guideline [30] denoted that many conditions or comorbidities are associated with the onset or development of HF and different patients may have different comorbidities, we can see that the important comorbidity for Patient 1 is *CHD,* while for Patient 2 diabetes is an important comorbidity. Besides, we can find that *CCB (calcium channel blocker)* is identified as one of the top-ranked predictor of readmission for Patient 1 who were prescribed *CCB*, which is in line with the guidelines. According to the guideline [30], *CCB* may be harmful and should be avoided to use in patients with low LVEF. The identified patient-specific risk factors could be further leveraged to assist the clinicians to customize the treatment strategies or intervention plans.

**Table 3:** The top-ranked features of the two randomly selected patients in Fig. 4 (attention weights> 0.2)

Patient 1			Patient 2		
Feature ID	Name	Attention weights	Feature ID	Name	Attention weights
21	NT-proBNP	0.114	21	NT-proBNP	0.192
32	Sodium	0.035	7	SBP	0.040
12	CHD	0.028	87	Left ventricular end-systolic volume	0.033
103	Spironolactone	0.027	15	Diabetes	0.028
89	Left ventricular end-diastolic volume index	0.025	60	Platelet count	0.028
94	CCB	0.024	–	–	–
78	Left atrial diameter	0.023	–	–	–

Moreover, the proposed model can not only provide individualized risk factors for each patient, but also provide the most frequent risk factors for all patient samples in a global sense. In Table 4, we listed the top-ten ranked features selected by both the proposed model and LR respectively. For the proposed model, the features are ranked by the frequency of the feature occurred in the top-ten features (ranking by attention weights) of each patient. For example, *NT-proBNP* is in the top-10 ranked features for all patients, while *CCB (calcium channel blocker)* is in the top-10 ranked features for 374 patients. With respect to LR, the selected patient features are ranked by the absolute values of the coefficients of these features in the learning LR model. It can be seen that there are three features are the same for the two models, i.e. *NT-proBNP, SBP (systolic blood pressure)* and *Platelet count,* which have been reported to be predictive for the prognosis of HF in literature [30]. E.g., hypertension may be the single most important modifiable risk factor for HF, whilst elevated levels of diastolic and especially systolic blood pressure are major risk factors for the development of HF [30]. The findings in [31] demonstrated an association between thrombocytopenia (low platelet count) and increased mortality in patients with heart failure.

**Table 4:** The top-ten globally ranked features of the proposed model and LR

MLP_attention			LR	
Feature ID	Name	Frequency	Feature ID	Name
21	**NT-proBNP**	736	21	**NT-proBNP**
94	CCB	374	7	**SBP**
78	Left atrial diameter	225	41	Lactate dehydrogenase
103	Spironolactone	180	57	Monocytes ratio
7	**SBP**	167	3	Height
32	Sodium	146	8	DBP (Diastolic blood pressure)
85	Interventricular septal thickness	146	5	BMI
87	Left ventricular end systolic volume	140	34	Phosphorus
10	Anemia	135	59	Basophil ratio
60	**Platelet count**	126	60	**Platelet count**

In addition to the three common risk factors, the proposed method identified three echocardiographic measurements in the top-ten ranked factors which were not identified by LR, i.e. *Left atrial diameter*, *Interventricular septal thickness* and *Left ventricular end systolic volume*. Echocardiography is used to evaluate the cardiac structure changes and left ventricular systolic function, and can help the clinicians make diagnosis and predict the risk of subsequent events (e.g. readmission) [30].

## Discussion

From the experimental results, we have some interesting findings as follows:

- Combining Tables 1 and 2, our proposed model outperformed the baseline models MLP and LR statistically. It demonstrates that the attention mechanism can improve the performance of neural networks.
- From Fig. 2, we can see that our proposed model achieved better performance with less number of layers (one layer) than MLP (four layers). Note that less number of layers of a deep neural work may have lower computational cost during training. In addition, it is interesting that the performance of the proposed model drops with the increasing of the number of hidden layers. We plan to investigate this phenomenon in our future work.
- The attention mechanism can identify the patient-specific features related to the outcomes, which can not only help the domain experts understand the prediction outcomes, but also support the decision makers to make individualized decisions.
- We can also obtain the important feature in the population level by counting the frequency of the top-ranked features in all patients.

It should be mentioned that there exist some limitations in this study needed to be investigated in the future. In our proposed model, the attention weights for all the features are positive, which is not able to tell us whether the influence of the feature is positive or negative like LR. We plan to work on this issue in our future work. In addition, the dataset used in this study is small, while the deep learning models usually need large volume of data for training. We plan to validate the proposed model on larger datasets.

## Conclusions

In this paper, we present an attention-based neural network model to improve the interpretability of the generated prediction results by the model. The patient-specific attention weights obtained from the model can not only help the clinicians understand the prediction outcomes, but also assist them to make further clinical decisions, such as customizing individualized treatment strategies or intervention plans for patients. We evaluated the proposed model on a real-world clinical dataset to address a specific clinical prediction problem, i.e., the readmission prediction for heart failure patients. The experimental results show that our proposed model outperforms the baseline models in terms of both accuracy and AUC.

Although our results have been encouraging in this study, the proposed approach could be further improved. At first, the dataset used in this study is relatively small, while the deep learning models usually need large volume of data for training. We plan to validate the proposed model on larger datasets in the future and compare the proposed model to more existing models. Besides, in this study, we simply exploited the numerical information for prediction. In our future work, we decided to utilize the abundant text data in the EHR (e.g. discharge notes, daily progress notes) to generate text interpretations.

## Data Availability

The datasets generated and/or analyzed during the current study are not publicly available due to the hospital’s regulations, but are available from the corresponding author on reasonable request.

## Abbreviations

LR: Logistic regression
MLP: Multilayer perceptron
HF: Heart failure
NT-proBNP: N terminal pro B type natriuretic peptide
cTnT: Cardiac troponin T
AUC: Area under ROC curve
CHD: Coronary heart disease
SBP: Systolic blood pressure
DBP: Diastolic blood pressure
